# Evaluating the efficacy and safety of single-agent etoposide intra-CSF chemotherapy in children and young people with relapsed/refractory central nervous system tumours

**DOI:** 10.1007/s00381-023-05872-w

**Published:** 2023-03-23

**Authors:** Anna Butler, Lisethe Meijer, Jo-Fen Liu, Manjit Chohan, Ibrahim Jalloh, Donald Macarthur, Margaret Parr, Sophie Wilne, Shaun Wilson, David Walker, Richard Grundy, Madhumita Dandapani

**Affiliations:** 1 Nottingham University Hospitals NHS Trust, Nottingham, NG7 2UH UK; 2 Department of Neuro-Oncology, Princess Maxima Centre for Paediatric Oncology, Utrecht, The Netherlands; 3 Children’s Brain Tumour Research Centre, University of Nottingham, Nottingham, NG7 2RG UK; 4 Cambridge University Hospitals NHS Trust, Cambridge, CB2 0QQ UK; 5 Oxford University Hospitals NHS Trust, Oxford, OX3 7LE UK

**Keywords:** Intra CSF etoposide, Chemotherapy, Central nervous system tumours, Relpase, Blood braian barrier, Progression-free survival

## Abstract

**Purpose:**

The aim of the project was to evaluate intra-CSF etoposide administration in a palliative setting for children and young people with relapsed/refractory central nervous system (CNS) tumours, with the primary endpoints being overall survival and progression-free survival time. A safety endpoint was to assess the side effect profile and complications of intra-CSF etoposide.

**Methods:**

Thirty-five patients under the age of 30 years (median age: 5.33 years) were enrolled onto the project. The cross-centre study was a service evaluation, with a data collection spreadsheet designed in Nottingham and completed by both Nottingham and Oxford centres. Data was analysed using SPSS, assessing the overall survival and progression-free survival times, as well as the 6-month and 1-year survival rates.

**Results:**

The median overall survival and progression-free survival times were 10.97 and 5.91 months, respectively. The 6-month and 1-year overall survival rates were 67% and 48%, and the progression-free survival rates were 50% and 22%. Age at the start of intra-CSF therapy was significantly associated with overall survival (*P* = 0.046), with the 6 + age group having improved overall survival. Treatment type was significantly associated with overall survival (*P* = 0.012), with etoposide intra-CSF treatment being associated with improved overall survival. Treatment duration was significantly associated with both overall survival (*P* < 0.001) and progression-free survival (*P* < 0.001).

**Conclusion:**

Intra-CSF etoposide treatment has shown to increase both overall and progression-free survival significantly, whilst having few side effects and maintaining a good quality of life for patients, reflecting it as a beneficial therapy in the palliative setting.

## Introduction

CNS tumours are the leading cause of death in childhood [1]. Leptomeningeal dissemination of CNS tumours can occur both in primary and more commonly in relapsed disease [2]. Leptomeningeal metastasis (LM) confers a grave prognosis at relapse with median survival for patients reported as between 4 weeks and 3 months in adult literature [3, 4], depending on the type of tumour.

Leptomeningeal dissemination of childhood CNS tumours is most commonly seen in medulloblastomas, as well as ependymomas, malignant gliomas, and germ cell tumours [5]. More than a third of children with medulloblastoma will have LM when first diagnosed, and over two-thirds will have disseminated disease if treatment is ineffective and they relapse [6]. The pattern of spread to the meninges and CSF also causes significant morbidity to the patients, with headache and cranial nerve involvement being prominent symptoms [7]. Data on outcomes for children with LM are limited to small studies, with a significant proportion of children with haematological malignancies, who are known to have better outcomes than those with primary CNS tumours [5]. Moreover, there is no consensus on how to treat children diagnosed with a CNS tumour who present with LM at relapse, with treatment options being limited due to the extensive multimodality of initial treatment.

One of the main challenges of treating LM is to overcome the blood–brain barrier. Intra-CSF chemotherapy involves delivering chemotherapy to tumours through the CSF, bypassing the blood–brain barrier, meaning drugs can be delivered directly to the brain [8]. It therefore uses a fraction of the dose used intravenously, and there are practically fewer systemic side effects due to very low drug levels in the blood [8]. Intra-CSF chemotherapy has been used to treat LM in haematological malignancies in frontline and relapse settings [9]. Less research has been done into the use of intra-CSF chemotherapy in brain tumours, but it is now being used in combination with other therapies to treat some CNS tumours, for example as an alternative to radiotherapy in infants, or as a palliative approach for patients with LM [8].

Intra-CSF delivery methods include the intraventricular route, which enables drugs to be delivered via an implanted device called an Ommaya reservoir to the lateral cerebral ventricle of the brain [10]. Intra-CSF therapy may also be administered by the intrathecal method, via a lumbar puncture procedure through a lumbar port [7, 10]. Intra-CSF therapy has been shown to have a relatively good safety profile in many studies, with patients suffering from only mildly adverse effects; however, particular attention must be paid during administration as there have been incidents of child deaths from errors of prescribing and preparation of chemotherapy [8]. Infection of the implanted device is one of the most common complications of intra-CSF therapy, with *Staphylococcus epidermidis* and *Staphylococcus aureus* being common causative organisms [10]. Non-infectious complications can include port malposition/migration which can cause obstruction, subcutaneous CSF leaks, and intracerebral haemorrhage in intraventricular devices [8, 10].

The first reported successful and safe administration of etoposide intraventricularly was by van der Gaast et al. in 1992 [11]. Etoposide is a derivative of podophyllotoxin that functions as a toposisomerase II inhibitor, and induces breaks in single and double strands of DNA, all of which prevents new DNA synthesis as well as causing cell death in tumour cells [12]. This mainly occurs in the G2 phase and S phase of the cell cycle, with its mode of action making it different from current chemotherapy agents used to treat LM [12]. Etoposide also has shown efficacy in the treatment of both solid tumours and haematological tumours, demonstrating it can act on both slow growing and rapidly proliferating tumours [13, 14].

Intraventricular etoposide is therefore a potential novel agent for the treatment of LM. Chamberlain et al. sought to explore this in their phase II trial evaluating the effectiveness of intraventricular etoposide in treating LM [14]. Twenty-seven patients with various primary tumour types, including 4 patients with brain tumours, were treated with intraventricular etoposide and concurrent radiotherapy or systemic chemotherapy in the study, with all having documented LM [14]. Seven of the patients showed a cytological response to the treatment, as well as stability or even improvement in neurological symptoms after 8 weeks of treatment [14]. However, 8 patients had progression of their disease during treatment, with 12 patients never having their CSF cleared of positive cells on cytological evaluation, although these patients had stability in neurological status clinically [14]. Minimal toxicity related to the treatment was observed, with no haematological side effects noted, and 5 cases of transient chemical arachnoiditis documented which were readily treated with oral steroids [14]. Overall, there was a progression-free survival rate of 11% at 6 months, and a 26% response to initial treatment over 8 weeks in all patients, which is encouraging compared to current treatment regimens for LM, suggesting intraventricular etoposide could be useful in combination therapy [14].

This prospective response to intraventricular etoposide was further seen in a pilot trial by Fleischhack et al., assessing the feasibility of giving the treatment to patients with relapsed or recurrent metastatic brain tumours [15]. Fourteen patients were eligible for the trial, aged 2.1 to 33.2 years, and were given systemic chemotherapy concurrently to the intraventricular etoposide [15]. Mild transient headache and infection occurred in 2 courses of treatment, with no haematological toxicity being observed [15]. Five of the patients showed clinical improvement after treatment in either neurological status or pain levels, 6 patients were clinically stable, and 3 patients had progressive disease [15]. Five of the patients also had positive cells in their CSF on cytological evaluation before the study; of the 4 of these able to have their CSF evaluated after treatment, clearance of the positive cells was demonstrated in all patients [15]. From the 9 patients with negative CSF cytology before the study, CSF remained negative in all patients whilst on treatment [15]. This shows an apparent clinical and cytological response to intraventricular etoposide in this group of patients [15]. However, in 2 patients who had regression of spinal metastases whilst on treatment, new parenchymatous lesions developed in the brain, displaying that intraventricular treatment may have insufficient penetration into the parenchyma [15, 16]. Pharmacokinetic analysis of etoposide levels in the CSF found that 2–10 × the concentration of the drug can be achieved by administering the drug intraventricularly rather than intravenously, providing evidence for increased cytotoxic activity LM through intraventricular therapy [15].

## Aims

Based on multiple research trials in the literature, Nottingham University Hospitals (NUH) NHS trust, along with researchers from the Children’s Brain Tumour Research Centre, started using intra-CSF etoposide therapy for children and young people with relapsed/refractory brain tumours. Treatment was offered to patients who had failed multiple lines of treatment, and was used in the palliative setting to improve overall survival. Oxford University Hospitals has adopted this treatment approach after discussion with practitioners in Nottingham. This paper reports the results of a retrospective study of intra-CSF etoposide treatment in a service evaluation cohort of 35 patients with LM, at two centres in the UK.

The aim of the project was to evaluate the intra-CSF etoposide administration in a palliative setting for children and young people with relapsed/refractory brain tumours, with the primary aims of the project being overall survival and progression-free survival time. Further aims were to understand which types of patient respond well to this treatment, and to assess if patients experienced any complications during treatment and explore the safety profile of intra-CSF etoposide.

## Methods

### Intra-CSF chemotherapy

Dosing of intra-CSF etoposide was individualised and decided by the paediatric neuro-oncology MDT/consultant. A local guideline for the use of intra-CSF etoposide in children and young people was created by the NUH Trust, which based etoposide dosing on the patient’s age (Table 1). Etoposide was administered via an Ommaya reservoir for intraventricular administration, or in patients with a ventricular shunt, a lumbar port was used for intrathecal administration.

**Table 1:** Dosing of intra-CSF etoposide

Dose (mg)		Frequency	Administration (weeks)	Rest period (weeks)
Induction				
Under 2 years	0.5	Daily for 5 days	2	1
2 years	0.75	Daily for 5 days	2	1
3 years and over	1	Daily for 5 days	2	1
Consolidation				
Under 2 years	0.5	Daily for 5 days	1	2
2 years	0.75	Daily for 5 days	1	2
3 years and over	1	Daily for 5 days	1	2

### Inclusion/exclusion criteria

Thirty-five patients under the age of 30 years with LM and relapsed disease were enrolled onto the study. LM was confirmed by the presence of malignant cells in the CSF on cytological examination, and the date of this diagnosis was recorded. Relapse was defined as progression of the disease on MRI, or testing positive for tumour cells in the CSF on cytological examination.

### Monitoring response and safety assessment

In the Nottingham cohort of patients, we were able to monitor response to intra-CSF treatment by evaluating the 1^st^ MRI scan the patient had 6–9 weeks after starting treatment, with either a complete response, partial response, stable disease (unchanged or < 25% improved/worsened), or progression of disease being recorded. All patients were assessed for any side effects during treatment, with all complications being documented in the clinical notes, and any haematological effects of treatment being noted on patients’ blood tests.

### Statistical analysis

Data was analysed using SPSS version 28.0 [17], to look at the median overall survival time and progression-free survival time in the cohort of patients, as well as the 6-month and 1-year survival rates. Relapses in disease were recorded for progression-free survival time, and the date the patient died (if applicable) was recorded for overall survival time, with both survival times being calculated from the date the patient started therapy. Descriptive statistics were used to summarise the distribution of key variables. The Kaplan–Meier method was used for survival analysis, and the difference in survival distributions was tested by the log-rank test. The impact of different factors on survival time was analysed — such as age at start of therapy, tumour type, type of intra-CSF therapy, and duration of treatment. The significance level for all analyses was set as 5%.

## Results

### Study population

The median age of the population was 5.33 years (range: 0.33–29.56 years). There were three patients in the age 18–30 category; all other patients were under 18 years of age. Primary tumour diagnoses are detailed in Table 2. Tumour diagnoses were unable to be further classified into molecular subtypes as the majority of these cases are historic and this information was not available. Twenty-seven patients received intra-CSF etoposide (primary aim of the project) and 8 patients received different intra-CSF therapies such as methotrexate, topotecan, and cytarabine, with all patients except for two receiving concurrent systemic chemotherapy.

**Table 2:** Patient characteristics

		Count	Column *N* %
Centre of origin	Nottingham	26	74%
	Oxford	9	26%
Gender	M	18	51%
	F	17	49%
Patient’s diagnosis	Medulloblastoma	12	34%
	Ependymoma	8	23%
	Pineoblastoma	5	14%
	ATRT	5	14%
	CNS Ewing sarcoma	1	3%
	Rhabdomyosarcoma	1	3%
	GBM	1	3%
	Choroid plexus carcinoma	1	3%
	B-cell ALL	1	3%
Age at start of therapy (years)	0–5	14	40%
	6 +	21	60%
Age at LM diagnosis (years)	0–5	18	51%
	6 +	17	49%
Age at start of therapy (years)	Mean	9.30	
	Median (range)	7.30 (0.21–29.65)	
Age at LM diagnosis (years)	Mean	8.56	
	Median (range)	5.33 (0.33–29.56)	
Type of ICSF therapy	Etoposide	27	77%
	Other	8	23%
Reason for stopping treatment	Progression	14	40%
	Other	5	14%
	Completed treatment	4	11%
	Infection	4	11%
	Dose increase	3	9%
	Change of treatment plan	3	9%
	Not stopped treatment	2	6%
Number of treatment cycles	Mean	14	
	Median	10	
Treatment duration (months)	Mean	3.86	
	Median (range)	2.92 (0.13–16.89)	

### Survival

The median overall survival time was 10.97 months, with a confidence interval of 5.43–16.52 ( Table 3). The median progression-free survival time was 5.91 months, with a confidence interval of 5.25–6.57. The 6-month and 1-year overall survival rates were 67% and 48%, respectively, whereas the 6-month and 1-year progression-free survival rates were 50% and 22%.

**Fig. 1:**
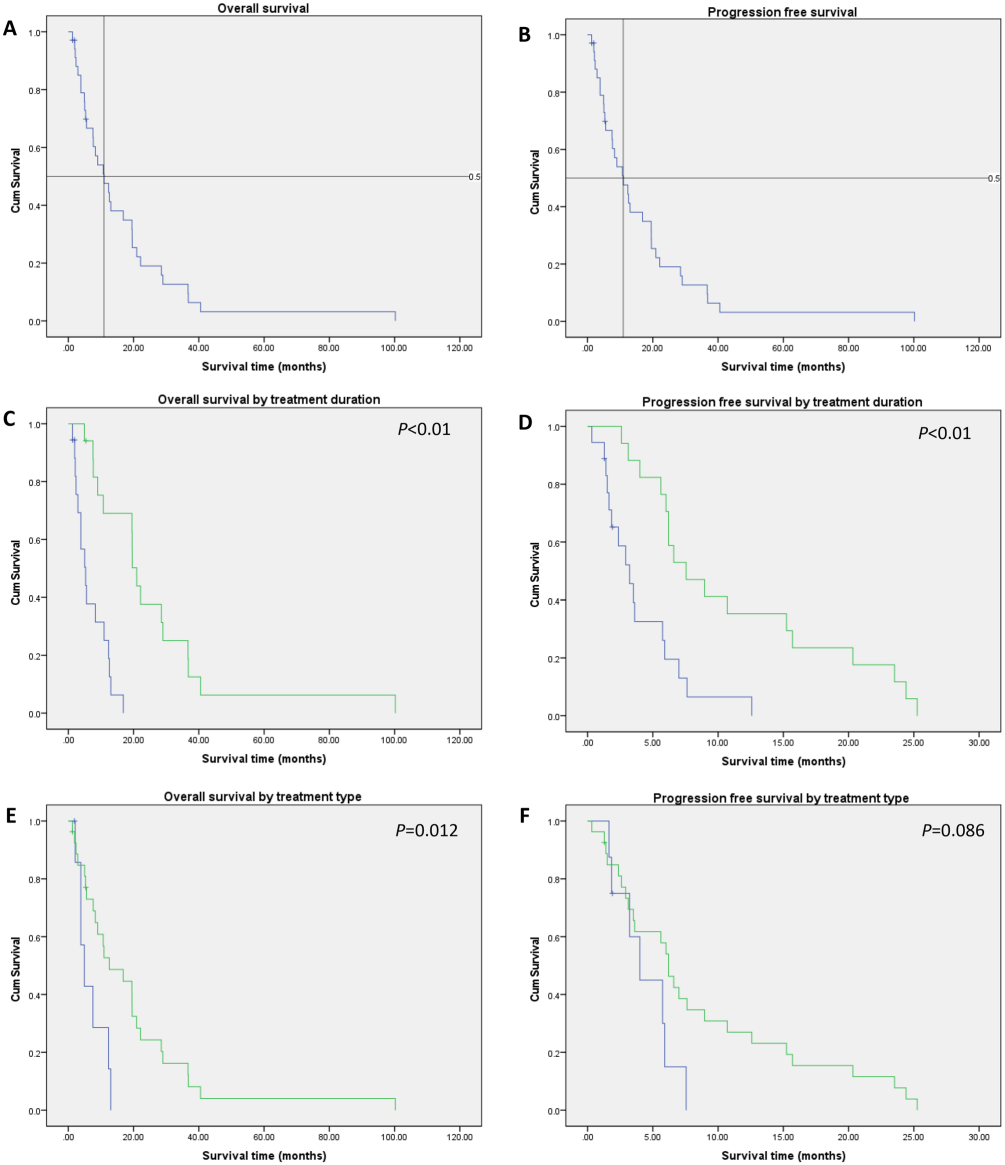
Kaplan–Meier curves of overall survival of all patients (**A**) and progression-free survival of all patients (**B**). The overall survival (**C**) and progression-free survival (**D**) by treatment duration, with 0–3 months of treatment (blue) and 3 + months of treatment (green) groups. The overall survival (**E**) and progression-free survival (**F**) by treatment type, grouped into etoposide intra-CSF treatment (green) and other intra-CSF treatment (blue)

Age at the start of intra-CSF therapy was significantly associated with overall survival (*P* = 0.046), with the 6 + age group having better overall survival (Fig. 1). Age at the start of intra-CSF therapy was not significantly associated with progression-free survival (*P* = 0.290). Type of primary tumour was not significantly associated with overall survival (*P* = 0.449) or progression-free survival (*P* = 0.524), when split into the groups of medulloblastoma, ependymoma, and all other types of tumour.

Treatment duration was significantly associated with both overall survival (*P* < 0.001) and progression-free survival (*P* < 0.001), when split into the groups of 0–3 months and 3 + months of treatment, with 3 + months being associated with improved survival. When split into the groups of 0–6 months and 6 + months, treatment duration was significantly associated with overall survival (*P* = 0.039), but not with progression-free survival (*P* = 0.195). Treatment type was significantly associated with overall survival (*P* = 0.012), with etoposide intra-CSF treatment being associated with improved overall survival. However, treatment type was not significantly associated with progression-free survival (*P* = 0.086).

**Table 3:** Overall and progression-free survival (months)

	Overall survival	Progression-free survival
Mean	16.51	7.77
Median	10.97	5.91
95% lower CL median	5.43	5.25
95% upper CL median	16.52	6.57
6-month survival	67%	50%
1-year survival	48%	22%

### Response to treatment

In the Nottingham cohort of patients, a complete response to initial intra-CSF treatment was noted on the 1^st^ MRI scan of 11 patients (42%) (Table 4). A partial response was noted in 7 patients (27%); however, no response was noted in 8 patients in total (31%). Out of these patients who did not respond, 2 patients had stable disease (8%), whereas 6 patients had progression of disease (23%).

**Table 4:** Initial response on 1^st^ MRI scan (6–9 weeks after starting treatment)

	Count	Column *N* %
Complete response	11	42%
Partial response	7	27%
No response — progression	6	23%
No response — stable disease	2	8%

### Complications

There were 9 cases (26% of patients) of device-related infection and 6 cases (17% of patients) of nausea or vomiting reported during treatment (Table 5). There were 4 cases of each of fatigue, febrile neutropenia, and headaches, and 3 cases of systemic infection. Anaemia and thrombocytopenia were each noted in two patients, which required blood and platelet transfusions, respectively, but did not delay treatment. There was one report each of hydrocephalus, neurological deficits, and seizures in this cohort (Table 5).

**Table 5:** Side effects and adverse events

	Count	Column *N* %
Device-related infection	9	26%
Nausea or vomiting	6	17%
Fatigue	4	11%
Febrile neutropenia	4	11%
Headaches	4	11%
Systemic infection	3	9%
Anaemia	2	6%
Thrombocytopenia	2	6%
Hydrocephalus	1	3%
Neurological deficits	1	3%
Seizures	1	3%
Meningitis/encephalitis	0	0%

## Discussion

This is the first reported study to analyse the effect of intra-CSF etoposide administration on both overall survival time and progression-free survival time, in a cohort of young patients suffering from relapsed or refractory brain tumours with LM. Intra-CSF etoposide improved overall and progression-free survival significantly, compared to historical data and other treatment regimens for LM. For example, the median overall survival time was calculated as almost 11 months, and progression-free survival time as 5.9 months, which is a considerable advance from the 3 weeks–4 months reported in the literature.

Furthermore, the 6-month and 1-year progression-free survival rates were calculated as 50% and 22%, respectively, showing an increase from previous trials evaluating the effectiveness of intra-CSF etoposide, with one trial reporting the 6-month progression-free survival rate as 11%. The aforementioned trial also reported a median progression-free survival time of 20 weeks; however, this only included the 26% of patients who responded to treatment [14].

Increased treatment duration was significantly associated with increased overall and progression-free survival (*P* < 0.001), with 3 + months of intra-CSF treatment being of particular benefit. This is not surprising however, as other confounding factors such as increasing disease burden causing the patient to deteriorate and stop treatment will have also contributed to this result. However, there was also a significant association in the 6 + months of the treatment group, suggesting that those patients which respond to treatment initially will continue to respond and benefit from ongoing intra-CSF etoposide chemotherapy.

Increased age at the start of treatment was also significantly associated with increased overall survival (*P* = 0.046), as the 6 + years age group was found to have improved overall survival when compared to the 0–5 years age group. This was an unexpected result, but must be cautiously interpreted due to the small sample size and diverse population, and when age at LM diagnosis was considered instead of age at start of treatment, no significant association was found.

Intra-CSF etoposide treatment was significantly associated with longer overall survival (*P* = 0.012), when compared to other intra-CSF treatments. This suggests intra-CSF etoposide may be superior to other therapies at treating LM in young patients with relapsed CNS tumours. Furthermore, this is a treatment that can be given in the outpatient setting as part of ambulatory care, with a low burden on patients’ quality of life compared to intensive relapse treatments. Patient narratives have highlighted this, with one patient who completely responded to treatment, stating they were given an “extra 2–3 years of life where they managed to travel” and another stating they had “no side effects whatsoever, you can’t even tell they’ve done it” after receiving treatment [18].

We found 42% of patients had a complete response to initial treatment, and a partial response was noted in 27% of patients, meaning over two-thirds of patients had some response to intra-CSF etoposide treatment in this cohort. Out of the patients with no clear response, 8% still had stability of disease, with 23% showing progression on the scan. However, the majority of these patients were clinically well, with little or no side effects resulting from treatment.

Overall, intra-CSF etoposide was found to be a safe and tolerable treatment, with the majority of patients experiencing minimal side effects or toxicity. The most common side effects were nausea/vomiting, headaches, and fatigue. The most common complication was device-related infection; however, all of these patients were readily treated with antibiotics and by removal of the device, with only one patient developing a systemic infection. Haematological toxicity was very minimal, with the two cases of febrile neutropenia with anaemia or thrombocytopenia thought to be attributable to concurrent systemic chemotherapy.

Sensory neurological deficits were observed in one patient, but it should be noted that the patient was additionally receiving three oral chemotherapy drugs during treatment. There were two cases of patients having to stop treatment due to hypothalamic dysfunction; however, intraventricular etoposide was likely only a contributing factor in one case, as the probable cause of the other case was a thalamic bleed post tumour surgery. The one reported episode of hydrocephalus was likely caused by increasing disease burden from tumour progression. This could have been the case in the one patient who started experiencing seizures; however, the patient was further noted to have undergone intensive systemic chemotherapy and radiotherapy.

This safety profile is comparable to the multiple trials for intraventricular etoposide documented in the literature, with no serious side effects being reported [14]. The response to treatment in this cohort is an improvement from the Chamberlain et al. trial, which found that 26% of patients responded to intraventricular etoposide within the first 8 weeks of treatment, with our study finding 42% of patients had a complete response [14]. This difference could be due to the range of different primary tumours studied and older age group in the Chamberlain et al. trial, whereas our study specifically looked at CNS tumours in younger patients. Moreover, the dose of intra-CSF etoposide that the patients received was much lower than the dose in our study, with patients receiving 0.5 mg for 5 days every 4 weeks during maintenance of treatment, compared to 0.5–1 mg of etoposide for 5 days every 3 weeks in our study [14].

This study emphasises the need for further research into the dosing of intra-CSF etoposide, to compare which dosing regimen improves survival the most, whilst maintaining a small number of side effects. More research into this treatment, using a larger number of patients from different centres, and studying robust neurocognitive outcomes, would further establish intra-CSF etoposide as a beneficial therapy in the palliative setting, and hopefully more centres would be encouraged to implement this treatment.

## Data Availability

The datasets generated during and/or analysed during the current study are available from the corresponding author on reasonable request.
